# 1,1′-(Diazene-1,2-diyl)bis(4-nitro-1H-1,2,3-triazole-5-carboxamide): An N8-Type Energetic Compound with Enhanced Molecular Stability

**DOI:** 10.3390/molecules30122589

**Published:** 2025-06-13

**Authors:** Moxin Sun, Wenjie Xie, Qi Lai, Gang Zhao, Ping Yin, Siping Pang

**Affiliations:** 1 School of Materials Science and Engineering, Beijing Institute of Technology, Beijing 100081, China; 3120221080@bit.edu.cn (M.S.); 3220211405@bit.edu.cn (W.X.); laiqi@bit.edu.cn (Q.L.); pangsp@bit.edu.cn (S.P.); 2 Sichuan Huachuan Industrial Co., Ltd., Chengdu 610106, China; 3 School of Materials Science and Engineering, Harbin Institute of Technology (Shenzhen), Shenzhen 518055, China

**Keywords:** long nitrogen chain compounds, nitrogen-rich heterocyclic ring, azo bridge, nitro-1,2,3-triazolecarboxamide, low-sensitivity energetic materials

## Abstract

The safety concerns associated with sensitivity issues regarding long nitrogen chain-based energetic compounds, especially for eight or more catenated nitrogen atoms in backbones, need to be resolved. Incorporating specific functional groups represents a key approach for enhancing stability in organic energetic materials. This study reports the synthesis of 1,1′-(diazene-1,2-diyl)bis(4-nitro-1H-1,2,3-triazole-5-carboxamide) (**S8**), an N8-chain compound featuring strategically placed amide groups. Employing THA(*O*-tosylhydroxylamine) and KMnO_4_, 1,1′-(diazene-1,2-diyl)bis(4-nitro-1H-1,2,3-triazole-5-carboxamide) (**S8**) was synthesized and underwent N-amination and oxidative azo coupling. Comprehensive characterization, including X-ray diffraction, mechanical sensitivity testing, and theoretical analysis, alongside comparative studies with known N8 compounds, revealed that S8 exhibits unprecedented stability within its class. Among reported N8-catenated nitrogen chain compounds, attributed to the incorporation of the amide functionality, **S8** demonstrates the highest impact sensitivity (IS = 10 J) and friction sensitivity (FS = 40 N) while maintaining excellent detonation performance (*D* = 8317 ms^−1^, *P* = 28.27 GPa). This work highlights the amide group as a critical structural part for achieving high stability in sensitive long-nitrogen-chain energetic materials without compromising performance.

## 1. Introduction

Energetic materials are regarded as a crucial part of advanced equipment which directly determines the development of civil applications and national defense. They are substances that can undergo intense oxidation–reduction reactions and release a large amount of energy under certain external stimuli. With the rapid development of energetic materials, higher requirements have been put forward for new energetic materials, including higher energy, greater security, and more environmental friendliness [1,2]. To meet these requirements, nitrogen-rich heterocycles like pyrazole, imidazole, triazole, tetrazole, and their derivatives have occupied a significant place in energetic materials in recent years [3], since they have a host of N–N and C–N bonds, exhibit high heats of formation, remarkable insensitivity, enhanced density, and environmental friendliness. Among them, long nitrogen chain heterocycles have aroused more interest due to their high heat of formation, which arises from the large energy release during the formation of N–N bonds [4]. However, materials with sufficient energy are often too sensitive to external mechanical stimuli. In contrast, many energetic materials with adequate stability do not meet the performance requirements [5]. Literature shows that long nitrogen chain compounds with both high energetic performance and good mechanical stability are rarely observed, and compounds become increasingly sensitive to external stimuli accompanied by the growth of the nitrogen–atom chain. According to the literature, the impact sensitivity of N6 is within the range of 2–14 J [6,7,8], the value of N8 is between 0.25 and 4 J [3,9,10,11], and the value of N10 is between 0 and 1 J [12].

Molecular design strategies that enhance electron delocalization and conjugation effects are critical for modifying material properties, as demonstrated in diverse chemical systems [13,14,15]. Notably, prior studies have not explored the introduction of functional groups incorporating both hydrogen-bond donors and acceptors (e.g., amides) into long-catenated nitrogen chain compounds to address their inherent instability. To address this gap, the amide group was strategically introduced into the N8-type long-nitrogen-chain molecule **S8**. Our experimental findings demonstrate that this strategic modification significantly enhances the stability of **S8**, establishing a promising approach to address sensitivity issues in long-nitrogen-chain energetic materials.

**Figure 1:**
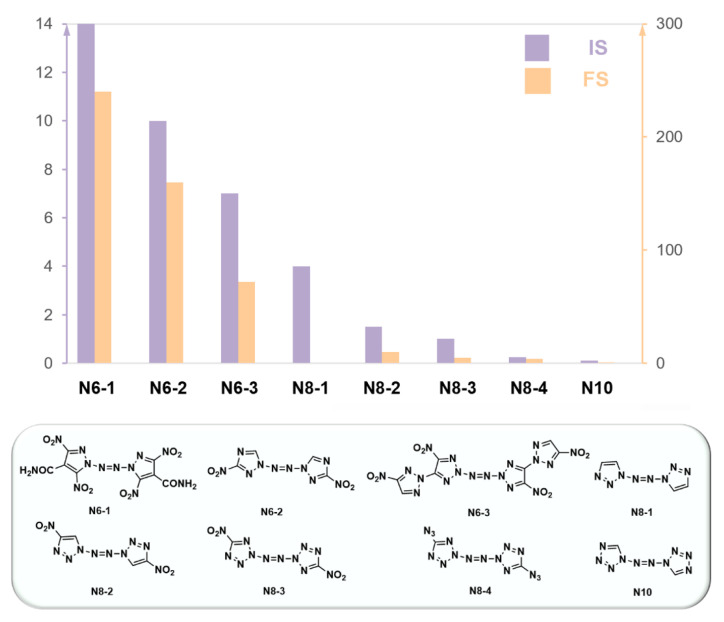
Mechanical sensitivity of long nitrogen chain compounds. N6-1 [8], N6-2 [7], N6-3 [6], N8-1 [4], N8-2 [9], N8-3 [10], N8-4 [11], N10 [12].

In this work, we report a simple method of HB introduction in triazole-based long nitrogen chain compound N8L to improve molecular stability. The compounds were characterized by infrared and multinuclear NMR spectroscopy. Additionally, single-crystal X-ray diffraction was performed to study the structural characteristics of intermolecular interactions (Figure 1).

## 2. Results and Discussion

### 2.1. Synthesis

**Scheme 1:**
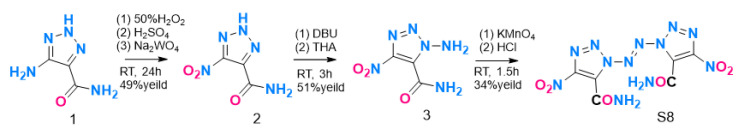
Synthetic route of compound **S8**.

The synthetic procedures for long-chain N-rich compounds are given in Scheme 1. Compounds **1** and **2** were prepared according to the literature [16]. Further reactions involving the neutralization of compound **2** with DBU and the amination of compound **2** using freshly prepared o-p-toluenesulfonyl-hydroxylamine (THA) gave rise to compounds **3** in 51% yields. It is worth noting that the reported reactions of 1,2,3-triazole have rarely shown regioselectivity [17]. For example, the amination of diammonium 4,4′-bis(5-nitro 1,2,3-2H-triazolate) led to the formation of three isomers [17], and the amination of 4-nitro-2H-1,2,3-triazole led to the formation of two isomers [9]. However, no isomers of compound **3** could be observed in this reaction, and the selective N-amination could be rationalized from the steric effect of the amide group.

### 2.2. Single Crystal X-Ray Structure Analysis

**Figure 2:**
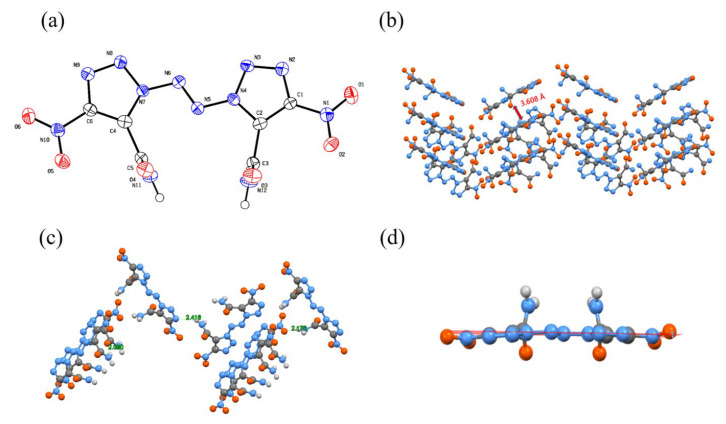
(**a**) Single-crystal structure of compound **S8**; (**b**) Crystal stacking diagram of compound **S8**; (**c**) Hydrogen bonding networks of compound **S8**; and (**d**) Planarity of the compound **S8**. (oxygen atoms (O) are represented by red spheres, nitrogen atoms (N) by blue spheres, and carbon atoms (C) by gray spheres. Hydrogen atoms (H) are depicted as small white spheres. All atoms are illustrated using the ball-and-stick model).

To confirm the structures of **S8** and explore their HB interactions, crystals suitable for single-crystal X-ray diffraction were obtained from the slow evaporation of chloroform solution. Compound **S8** crystallizes in the monoclinic space group P2_1_2_1_2_1_ with a calculated crystal density of 1.763 g cm^−3^ at 100 K (Z = 4). The crystal packing of S8 can be viewed as a face-to-face wavelike stacking with a 3.608 Å interlayer spacing, within the range of π–π interaction (<4.0 Å) [13] (Figure 2b). The packing index is 70.44%, which is a desirable packing type that contributes to low sensitivity. The bond lengths of C6-NO2 and C1-NO2 are 1.448(6) and 1.427(6) Å, respectively. There are four molecules in the **S8** unit cell, and each molecule has a near-planar geometry, which can be further confirmed by the torsion angles of [N7-N8-N9-C6 = −0.1 (5)°, N5-N4-N3-N2 = 179.7 (4)°, C2-N4-N3-N2 = 0.3(6)°, N3-N2-C1-N1 = 179.8(4)°], and the torsion angles of the nitro group (C6-NO2)[O5-N10-C6-C4 = 8.6(8)°], (C1-NO2)[C2-C1-N1-O1 = −11.7(7)°].

**Figure 3:**
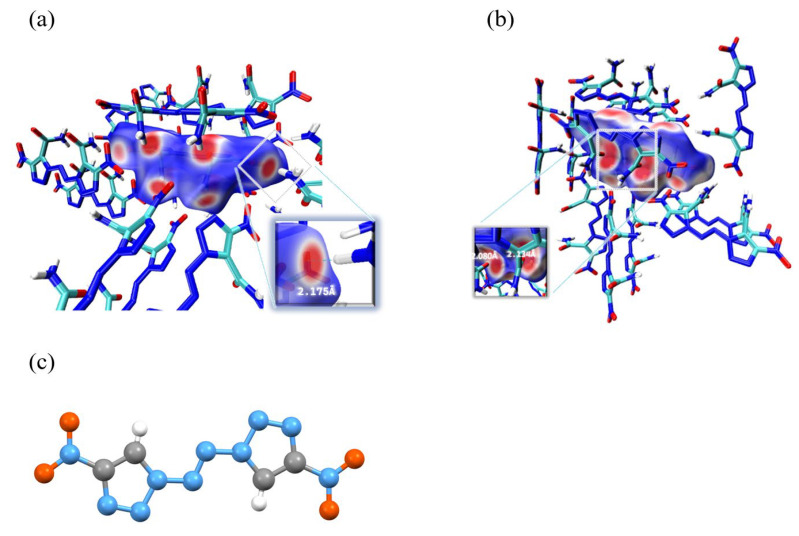
Hirshfeld surfaces for compound **S8** (**a**,**b**); Given the very similar structure. In subsequent chapters, frequent comparative analysis was conducted on **S8** and **N8L** to observe whether their performance was improved. The structure of **N8L** is shown in (**c**). (oxygen atoms (O) are represented by red spheres, nitrogen atoms (N) by blue spheres, and carbon atoms (C) by gray spheres. Hydrogen atoms (H) are depicted as small white spheres. All atoms are illustrated using the ball-and-stick model.)

X-ray single-crystal analysis revealed that each **S8** molecule participates in eight strong intermolecular hydrogen bonds toward the nitrogen of the triazole ring, oxygen and hydrogen of the amide, and the oxygen of the nitro group (Figure 3). Red spots on the **S8** Hirshfeld surface (Figure 3a) are large and colorful, indicating that the intermolecular hydrogen bonds in **S8** are plentiful and strong. Each molecule within the crystal structure participates in 8 intermolecular hydrogen bonds. The above analysis shows 3D HB networks on **S8**.

**Figure 4:**
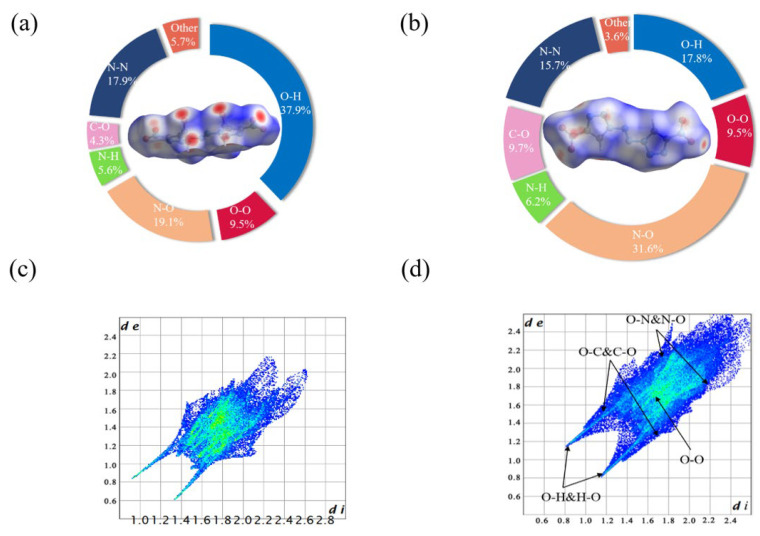
Quantitative analysis of intermolecular hydrogen bonds of **S8** and **N8L**. Hirshfeld [18] surfaces (inside) and p ie graphs of **S8** (**a**) and **N8L** (**b**), respectively, showing the percentage contribution of the individual atomic contacts to the Hirshfeld surfaces (**c**,**d**). Highlighting N…H and O…H contacts (inside) and 2D-fingerprint plots in crystal stacking, respectively.

Two-dimensional fingerprints of **S8** and the associated Hirshfeld surface were analyzed. Red spots in **S8** are more extensive than those of **N8L**, indicating that the intermolecular hydrogen bonds in **S8** are stronger and more plentiful than those of **N8L**. Red spots in **S8** are on the same side of the molecular, which indicates that the intermolecular interaction mainly occurs on the side with the functionalized group. **S8** possesses strong hydrogen-bonding interactions, which can be rationalized from the strong O…H spikes in the Hirshfeld surface (Figure 4c). Quantifying this in Figure 4a,b, 43.5% of interactions in **S8** are N…H and O…H bonds, while for **N8L,** this value is only 24%.

**Figure 5:**
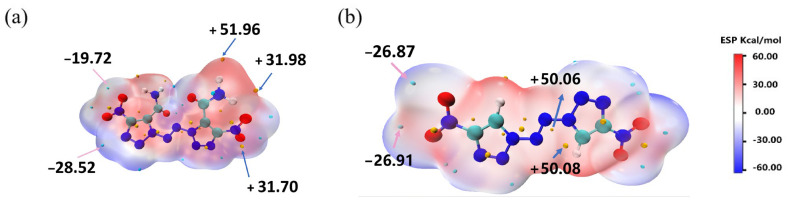
Electrostatic potential surfaces (ESPs) for **S8** (**a**) and **N8L** (**b**), respectively. The blue and red spheres in the diagram represent the surface local minima and maximum of ESP, respectively.

Electrostatic potential surfaces (ESPs) were calculated to compare the difference in physicochemical properties [19,20,21]. The maximum value of **N8L** (+53.72 kcal mol^−1^) is higher than that of **S8** (+51.96 kcal mol^−1^). It is worth noting that ESP values may be related to mechanical sensitivities [22,23]. The number of **S8** is 80.48 kcal mol^−1^, which is larger than that of **N8L** (79.91 kcal mol^−1^) (Figure 5).

**Figure 6:**
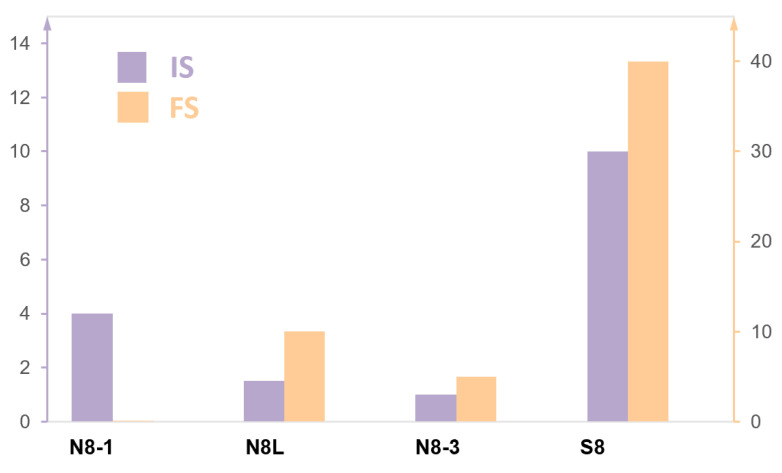
Comparison of sensitivity (Friction sensitivity and impact sensitivity) between **S8** and compounds **N8-1** [4], **N8L** [9], **N8-3** [10].

To investigate the influence of the introduction of amide droup, the density, detonation performance, thermal stability, mechanical sensitivity, and heat of formation of **S8** were studied and compared with other N8 compounds. We tested the mechanical sensitivity of **S8** using the BFH 10 model BAM drop hammer impact sensitivity apparatus and FKSM 10BAM model BAM friction sensitivity apparatus at room temperature. The impact sensitivities (*IS*) and friction sensitivities (*FS*) of **S8** were *IS* = 10 J, and *FS* = 40 N, which is much less sensitive than that of other N8-type energetic compounds. According to the study of crystal structures and comparison with **N8L**, we propose that the introduction of amide is responsible for the improvement in the mechanical sensitivity towards external mechanic stimuli. Other reported long-catenated nitrogen structures are listed below, and detailed sensitivity and detonation performance are drawn in Figure 6.

Based on isodesmic reactions and calculated by the Gaussian 09 D.01 program, **S8** has a fine heat of formation of 687.45 kJ mol^−1^, which can be attributed to the large number of N=N and N-N bonds in the molecule. Measuring by Gas pycnometers, the density of **S8** in 100k was 1.767 g cm^−3^. Key parameters were selected to calculate with EXPLO5 (version 6.05) to estimate the detonation performances. As a result, **S8** has a high detonation velocity and pressure with the calculated number of 8317 ms^−1^ and 33.43 GPa, respectively. Differential scanning calorimetry and thermal gravimetric results reveal that **S8** possesses higher thermostability, with a *T*_d_ of 177.6 °C. It is worth noting that the introduction of amide causes an improvement of 1 °C in thermal stability. It is also worth noting that this system represents the first example of the introduction of the amide group to improve molecular stability in the field of long nitrogen chain compounds (Table 1).

## 3. Materials and Methods

**Caution!** The novel compounds investigated in this work possess inherent energetic properties, creating a potential for detonation under specific external stimuli. Consequently, all experimental procedures involving these materials demand enhanced safety protocols. Prudent measures include the consistent use of protective shields, hearing protection (earplugs), safety goggles, and cut-resistant gloves.

### 3.1. Reagents and Instruments

**Reagents:** 3-Amino-3-iminopropanamide hydrochloride (1:1), sodium methoxide, p-toluenesulfonyl azide, sodium tungstate dihydrate, 50% hydrogen peroxide solution, dichloromethane, anhydrous sodium sulfate, ethyl acetate, triethylamine, ethyl acetohydroxamic acid, p-toluenesulfonyl chloride, perchloric acid, potassium permanganate, concentrated hydrochloric acid and acetonitrile all of analytical grade, were purchased from Shanghai Aladdin Biochemical Technology Co., Ltd, Beijing, China. Energy-chemical, Shanghai, China. Beijing Chemical Plant Co., Ltd. Beijing, China, Beijing Tong Guang Fine Chemcials Company, Beijing, China. Shanghai Meiruier Biochemical Technology Co., Ltd, Shanghai, China.

**Instruments:** AVANCE DRX-500 NMR scanner, Bruker, Fällanden, Switzerland; RCT basic magnetic stirrer, IKA Staufen im Breisgau, Germany; NI 10 Infrared Spectrometer, Thermo Fisher Scientific, Waltham, MA, USA; Q2000 Differential Thermal Scanner, US TA, New Castle, DE, USA; FLASH 2000 CHNS/O Element Analyzer, Thermo Fisher Scientific; BFH 10 BAM Drop Hammer Impact Sensitivity Instrument, FKSM 10 BAM Friction Sensitivity Tester, Edison Company, Beijing, China.

### 3.2. Computational Methods

- Electrostatic potential (ESP) and Heat of Formation

The geometric optimization of compounds **3** and **S8** was carried out using Gaussian 09, density functional theory (DFT), and the B3LYP/6-31g (d, p) basis set. The vibration analysis of the optimized structures showed no imaginary frequencies, indicating that these structures are at their minimum values on their respective potential energy surfaces. Subsequently, the electrostatic potentials of compounds **3** and **S8**, as well as the enthalpy of formation of compound **S8**, were calculated and analyzed using the B3LYP/6-31+g (d, p) basis set.

### 3.3. Compound 1,2 Prepared According to the Literature [25]

- Synthesis of compound **1**:

Malonamamidine hydrochloride (0.1 mol, 41.28 g) was added to a sodium methoxide solution (3 N, 100 mL) at 0 °C for reaction. After the neutralization reaction, the precipitate was isolated by filtering the final mixture and washed with ethanol (40 mL). To this filtrate, an ethanol solution of p-methylbenzenesulfonyl azide was added dropwise, and the mixture was stirred overnight at ambient temperature. After the reaction, the mixture was filtered, and the precipitate was washed with ethanol and then air-dried. Yield: white solid (80%). ^1^H NMR (d_6_-DMSO): δ7.40 (s, 1 H), 7.08 (s, 1 H), 5.79 (s, 2 H) ppm. ^13^C NMR (d_6_-DMSO): δ 157.5, 147.3, 137.5, 129.4 ppm. IR (KBr): ṽ:3392, 1690, 1672, 1535, 1340, 1130, 1002, 850, 568 cm^−1^. Elemental analysis of C_3_H_5_N_5_O (127.05): Calculated (%) C 28.35, H 3.97, N 55, O 12.59; found (%): C 28.01, H 4.10, N 54.5, O 13.39.

- Synthesis of compound **2**:

At 0 °C, Sodium tungstate dihydrate (6.5 g, 22.5 mmol) was dissolved in 50% H_2_O_2_ (100 mL), and concentrated sulfuric acid (0.5 mL) was added dropwise (the solution color is yellow at this time). Maintain the mixture at 0–10 °C, and the above 5-amino-1,2,3-triazol-4-formamide (3.14 g, 24.7 mmol) was added. After stirring for s days at ambient temperature, the mixture was filtered, and the precipitate was washed with H_2_O and dried in air. Yield: yellow solid (49%). ^1^H NMR (d^6^-DMSO): δ8.27 (s, 1 H), 8.16 (s, 1 H) ppm. ^13^C NMR (d_6_-DMSO): δ 163.5, 151.7, 111.4, 108.5 ppm. IR (KBr): ṽ: 3452, 2980, 1686, 1597, 1498, 1416, 1402, 1355, 1305, 1277, 1183, 953, 849 cm^−1^. Elemental analysis of C_3_H_3_N_5_O_3_ (157.02): Calculated (%) C 22.94, H 1.93, N 44.58, O 30.55; found (%): C 23.12, H 1.95, N 44.26, O 30.67.

- Synthesis of compound **3**:

Compound 2 (1570 mg, 10 mmol) was dissolved in 1,8-Diazabicyclo [5.4.0] undec-7-ene (1.57 mL, 10 mmol) at ambient temperature. After stirring for 30 min, 8 mL of freshly prepared O-Tosylhydroxylamine was added dropwise to this solution. After stirring for 3 h at ambient temperature, the mixture was filtered, and the precipitate was washed with H_2_O and dried in air. Purification was performed by column chromatography using a petroleum ether/ethyl acetate mixture (1:2, *v*/*v*) as the eluent. (40%). ^1^H NMR (d_6_-DMSO) δ 8.360 (s, 2 H), 7.509 (s, 2 H). ^13^C NMR (d_6_-DMSO) δ 156.97, 147.01, 129.95 ppm. IR (KBr): ṽ:3272, 1694, 1526, 1387, 1262, 1201, 1031, 1011, 846, 793, 715, 683, 666, 528, 472 cm^−1^. Elemental analysis of C_3_H_4_N_6_O_3_ (172.03): Calculated (%) C 20.94, H 2.34, N 48.83, O 27.89; found (%): C 21.12, H 2.22, N 48.69, O 27.97.

- Synthesis of compound **S8**:

Compound 3 (344 mg, 2 mmol) was added to a round-bottomed flask with 5 ml hydrochloric acid and 16 mL of KMnO_4_ (500 mg, 3 mmol) solution was added dropwise to this solution at ambient temperature. After stirring for 1.5 h at ambient temperature, the mixture was filtered, and the precipitate was washed with H_2_O and hydrochloric acid, then dried in the air. Yield: white solid (34%) ^1^H NMR (d_3_-CD_3_CN.): δ 7.133 (br, 2H), δ 7.045 (s, 2H). ^13^C NMR (d_3_-CD_3_CN): δ 181.296, 155.307, 130.311 ppm. IR (KBr): ṽ:1703, 1526, 1305, 908, 844, 831, 555, 533, 504 cm^−1^. Elemental analysis of C_6_H_4_N_12_O_6_ (340.04): Calculated (%) C 21.18, H 1.19, N 49.41, O 28.22; found (%): C 20.96, H 1.38, N 49.01, O 28.65.

### 3.4. Single-Crystal Preparation Method of 3 and S8

Single crystals of novel compounds **3** and **S8** were grown using the solvent evaporation method. A single crystal measuring 0.19 mm × 0.18 mm × 0.16 mm was selected to ensure the acquisition of high-quality diffraction data during the X-ray diffraction analysis. Data collection: APEX-III; cell refinement: SAINT V8.40A (Bruker, 2019); data reduction: SAINT V8.40A (Bruker, 2019); program(s) used to solve structure: SHELXT 2014/5 (Sheldrick, 2014, Nairobi, Kenya); program(s) used to refine structure: SHELXL2019/2 (Sheldrick, 2019). Supplementary Materials provide detailed information on crystallography. Subsequent analysis of the spatial configurations was performed using visualization software to elucidate the structure–property relationships.

### 3.5. Thermal Performance and FTIR Experimental Methods

- Differential scanning calorimetry experiments

For DSC measurements, samples (~0.5 mg) were accurately weighed using an analytical balance, loaded into hermetically sealed aluminum pans, and analyzed under a dry nitrogen purge flow of 20 mL·min^−1^. The temperature was ramped from 40 °C to 400 °C at a heating rate of 10 °C·min^−1^.

- In situ FTIR experiments

Fourier-transform infrared (FT-IR) spectra were obtained at 25 °C using a PerkinElmer Spectrum BX spectrometer equipped with an attenuated total reflection (ATR) accessory.

## 4. Conclusions

The stabilization of high-energy materials enables them to function across multiple technological domains. Through enhanced mechanical sensitivity, these advanced formulations allow for reliable deployment in extreme environments such as Arctic/desert combat zones. Most importantly, such research contributes to the development of the next generation of high-energy insensitive explosives. This study successfully synthesized **S8**, an N8-type long-nitrogen-chain compound. Experimental results and data analysis demonstrate that the incorporation of an amide group enhances the stability of long-nitrogen-chain molecules. **S8** exhibits the highest stability among all reported N8-type long-nitrogen-chain compounds to date. This work provides deeper insights into the relationship between molecular structure and performance. Furthermore, it offers valuable perspectives for designing energetic materials with superior properties, particularly low sensitivity.

## Figures and Tables

**Table 1:** The physical properties of compounds **S8** compared with other N8 compounds.

Comp.	*T*_d_ ^a^ [°C] ^a^	*d*^b^ [gcm^−3^] ^b^	Δ_f_*H* ^c^ [KJ mol^−1^] ^c^	*IS*^d^ [J] ^d^	*FS*^e^ [N] ^e^	*D*^f^ [ms^−1^] ^f^	*P*^g^ [GPa]
**N8L**	177	1.78	877.7	1.5	10	8916	33.5
**S8**	177.6	1.76	687.45	10	40	8317	28.27
**N8-1** ^h^	193.8	1.62	962	4	-	7764	25.24
**N8-3** ^i^	-	1.80	1153	<<<<1	<<<<5	9184	39
**N8-4** ^j^	114	1.81	1700	<<0.25	<<0.1	9515	36.6
**RDX**	204	1.80	70.3	7.4	120	8762	18.4
**TNT**	295	1.65	69.75	2.5–4	0.1–1	7190	34.9

^a^ Thermal decomposition temperature, °C. ^b^ Crystal density at 296 K, g cm^−3^. ^c^ Heats of formation (computed by the Gaussian 09 D.01 program [24]), kJ mol^−1^. ^d^ Impact sensitivity, J. ^e^ Friction sensitivity, N. ^f^ Detonation velocity (calculated with Explo5 v6.02). ^g^ Detonation pressure, GPa. ^h^ Ref. [4]. ^i^ Ref. [10]. ^j^ Ref. [11].

## Data Availability

CCDC 2362107 (**S8**), 2362106 (**3**) contain the supplementary crystallographic data for this paper. These data can be obtained free of charge via www.ccdc.cam.ac.uk/data_request/cif (accessed on 1 June 2025), or by emailing data_request@ccdc.cam.ac.uk, or by contacting The Cambridge Crystallographic Data Centre, 12 Union Road, Cambridge CB2 1EZ, UK; fax: +44 1223 336033.

## Supplementary Materials

The following supporting information can be downloaded at: https://www.mdpi.com/article/10.3390/molecules30122589/s1, Figure S1: Isodesmic reactions for **S8**; Table S1: Crystal data for compounds **3**; Table S2: Refinement for compounds **3**; Table S3: Data collection for compounds **3**; Table S4: Fractional atomic coordinates and isotropic or equivalent isotropic displacement parameters (Å^2^) for compound **3**; Table S5: Atomic displacement parameters (Å^2^) for compound **3**; Table S6: Geometric parameters (Å^2^) for compound **3**; Table S7: Crystal data for compounds **S8**; Table S8: Refinement for compounds **S8**; Table S9: Data collection for compounds **S8**; Table S10: Fractional atomic coordinates and isotropic or equivalent isotropic displacement parameters (Å^2^) for compound **S8**; Table S11: Atomic displacement parameters (Å^2^) for **S8**; Table S12: Geometric parameters (Å^2^) for **S8**; Figure S2: ^1^H NMR spectrum of compound **3** in DMSO-*d6*; Figure S3: ^13^C NMR spectrum of compound **3** in DMSO-*d6*; Figure S4: ^1^H NMR spectrum of **S8** in *d3*-CD_3_CN; Figure S5: ^13^C NMR spectrum of **S8** in *d3*-CD_3_CN; Figure S6: IR spectrum of compound **3**; Figure S7: IR spectrum of compound **S8**; Figure S8: DSC curve of compound **S8** at 10 °C min^−1^ [26,27,28].
