# Supplementary material for: 1,1′-(Diazene-1,2-diyl)bis(4-nitro-1H-1,2,3-triazole-5-carboxamide): An N8-Type Energetic Compound with Enhanced Molecular Stability
Source: Molecules. 2025 Jun 13;30(12):2589. doi: 10.3390/molecules30122589 (PMC12195902; doi:10.3390/molecules30122589)
Supplement: Supplementary file 1 [file molecules-30-02589-s001.zip › molecules-3539086-supplementary.pdf]

molecules

Supporting Information

1,1′-(Diazene-1,2-diyl)bis(4-nitro-1H-1,2,3-triazole-5-carboxamide): An N8-Type Energetic Compound with Enhanced Molecular Stability

Moxin Sun, Wenjie Xie, Qi Lai, Gang Zhao, Ping Yin and Siping Pang

Table of Contents

1. Computational Details....................S2
2. Detailed analysis of the crystal structure for compounds 3 and S8....................S2
3. ^{1}H and ^{13}C NMR spectra for all new compounds....................S11
4. IR spectra of all new compounds....................S13
5. DSC curves of the title compounds....................S14

References....................S10

1. Computational Details

Theoretical calculations were performed by using the Gaussian 09 suite of programs.[2] Gas phase heats of formation of the title compounds were computed based on an isodesmic reaction. The isodesmic reaction processes, that is, the number of each kind of formal bond is conserved, were used with the application of the bond separation reaction (BSR) rules. The molecule was broken down into a set of two heavy-atom molecules containing the same component bonds. The isodesmic reactions used to derive the HOF are shown in Fig. S1.

The enthalpy of the reaction was carried out by combining the M062X/6-311++G** energy difference for the reactions, the scaled zero-point energies (ZPE), values of thermal correction (HT), and other thermal factors. The solid-state heat of formation was further obtained by employing Trouton's rule according to equation 1 (T represents either melting point or decomposition temperature when no melting occurs prior to decomposition).[3]

$\Delta H_{sub} = 188/J\ mol^{-1}\ K^{-1} \times T$ (1)

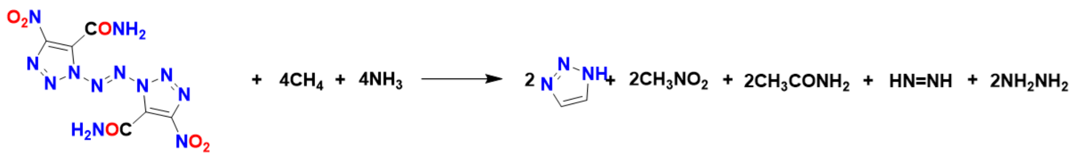

Figure S1. Isodesmic reactions for S8.

2. Detailed analysis of the crystal structure for compounds 3 and S8

Table S1 Crystal data for compounds **3**
$C_3H_4N_6O_3$
M_r = 172.12
Monoclinic, $P2_1/n$
a = 8.384 (4) Å
b = 6.987 (4) Å
c = 12.082 (6) Å
β = 108.259 (6)°
V = 672.1 (6) Å^3
F(000) = 352
D_x = 1.701 Mg m^{-3}
Mo $K\alpha$ radiation, λ = 0.71073 Å
Cell parameters from 2805 reflections
θ = 2.9–27.5°
μ = 0.15 mm^{-1}
T = 296 K
Block, colourless
0.19 × 0.18 × 0.16 mm

Table S2 Refinement for compounds **3**

Refinement on F^2
Least-squares matrix: full
$R[F^2 > 2\sigma(F^2)] = 0.035$
$wR(F^2) = 0.096$
$S = 1.06$
1518 reflections
118 parameters
0 restraints
Hydrogen site location: mixed
H atoms treated by a mixture of independent and constrained refinement
$w = 1/[\sigma^2(F_o^2) + (0.0559P)^2 + 0.1308P]$ where $P = (F_o^2 + 2F_c^2)/3$
$(\Delta/\sigma)_{max} < 0.001$
$\Delta\rangle_{max} = 0.21$ e Å^{-3}
$\Delta\rangle_{min} = -0.20$ e Å^{-3}
Extinction correction: *SHELXL2019*/2 (Sheldrick 2019), $Fc^*=kFc[1+0.001xFc^2\lambda^3/\sin(2\theta)]^{-1/4}$
Extinction coefficient: 0.174 (13)

Table S3 Data collection for compounds **3**

Bruker D8 VENTURE diffractometer
Absorption correction: multi-scan BRUKER;*SADABS*;2016; _exptl_absorpt_special_details _diffrn_ambient_temperature 296(2) _diffrn_radiation_wavelength 0.71073 _diffrn_radiation_type MoKα _ diffrn_source _diffrn_measurement_device_type 'Bruker D8 VENTURE'_diffrn_measurement_method _diffrn_detector_area_resol_mean _diffrn_reflns_number 3883 _diffrn_reflns_av_unetI/netI 0.0283 _diffrn_reflns_av_R_equivalents 0.0306 _diffrn_reflns_limit_h_min -10 _diffrn_reflns_limit_h_max 7 _diffrn_reflns_limit_k_min -9 _diffrn_reflns_limit_k_max 8 _diffrn_reflns_limit_l_min -15 _diffrn_reflns_limit_l_max 15 _diffrn_reflns_theta_min 2.617 _diffrn_reflns_theta_max 27.547 _diffrn_reflns_theta_full 25.242 _diffrn_measured_fraction_theta_max 0.980 _diffrn_measured_fraction_theta_full 0.989 _diffrn_reflns_Laue_measured_fraction_max 0.980 _diffrn_reflns_Laue_measured_fraction_full 0.989 _diffrn_reflns_point_group_measured_fraction_max 0.980 _diffrn_reflns_point_group_measured_fraction_full 0.989 _reflns_number_total 1518 _reflns_number_gt 1382 _reflns_threshold_expression 'I > 2σ(I)' _reflns_Friedel_coverage 0.000 _reflns_Friedel_fraction_max . _reflns_Friedel_fraction_full . _reflns_special_details
T_{min} = 0.453, T_{max} = 0.562
3883 measured reflections
1518 independent reflections
1382 reflections with $I > 2\sigma(I)$
Absorption correction: multi-scan BRUKER;*SADABS*;2016; _exptl_absorpt_special_details _diffrn_ambient_temperature 296(2) _diffrn_radiation_wavelength 0.71073 _diffrn_radiation_type MoKα _ diffrn_source _diffrn_measurement_device_type 'Bruker D8 VENTURE'_diffrn_measurement_method _diffrn_detector_area_resol_mean _diffrn_reflns_number 3883 _diffrn_reflns_av_unetI/netI 0.0283 _diffrn_reflns_av_R_equivalents 0.0306 _diffrn_reflns_limit_h_min -10 _diffrn_reflns_limit_h_max 7 _diffrn_reflns_limit_k_min -9 _diffrn_reflns_limit_k_max 8 _diffrn_reflns_limit_l_min -15 _diffrn_reflns_limit_l_max 15 _diffrn_reflns_theta_min 2.617 _diffrn_reflns_theta_max 27.547 _diffrn_reflns_theta_full 25.242 _diffrn_measured_fraction_theta_max 0.980 _diffrn_measured_fraction_theta_full 0.989 _diffrn_reflns_Laue_measured_fraction_max 0.980 _diffrn_reflns_Laue_measured_fraction_full 0.989 _diffrn_reflns_point_group_measured_fraction_max 0.980 _diffrn_reflns_point_group_measured_fraction_full 0.989 _reflns_number_total 1518

_reflns_number_gt 1382 _reflns_threshold_expression 'I > 2σ(I)' _reflns_Friedel_coverage 0.000 _reflns_Friedel_fraction_max . _reflns_Friedel_fraction_full .
_reflns_special_details

T_{min} = 0.453, T_{max} = 0.562

3883 measured reflections

1518 independent reflections

1382 reflections with $I > 2\sigma(I)$

Absorption correction: multi-scan
BRUKER;*SADABS*;2016;
_exptl_absorpt_special_details _diffrn_ambient_temperature 296(2)
_diffrn_radiation_wavelength 0.71073 _diffrn_radiation_type MoKα _
diffrn_source _diffrn_measurement_device_type 'Bruker D8
VENTURE'_diffrn_measurement_method _diffrn_detector_area_resol_mean
_diffrn_reflns_number 3883 _diffrn_reflns_av_unetI/netI 0.0283
_diffrn_reflns_av_R_equivalents 0.0306 _diffrn_reflns_limit_h_min -10
_diffrn_reflns_limit_h_max 7 _diffrn_reflns_limit_k_min -9 _diffrn_reflns_limit_k_max 8
_diffrn_reflns_limit_l_min -15 _diffrn_reflns_limit_l_max 15 _diffrn_reflns_theta_min 2.617
_diffrn_reflns_theta_max 27.547 _diffrn_reflns_theta_full 25.242
_diffrn_measured_fraction_theta_max 0.980 _diffrn_measured_fraction_theta_full 0.989
_diffrn_reflns_Laue_measured_fraction_max 0.980
_diffrn_reflns_Laue_measured_fraction_full 0.989
_diffrn_reflns_point_group_measured_fraction_max 0.980
_diffrn_reflns_point_group_measured_fraction_full 0.989 _reflns_number_total 1518
_reflns_number_gt 1382 _reflns_threshold_expression 'I > 2σ(I)' _reflns_Friedel_coverage 0.000 _reflns_Friedel_fraction_max . _reflns_Friedel_fraction_full .
_reflns_special_details

T_{min} = 0.453, T_{max} = 0.562

3883 measured reflections

1518 independent reflections

h = -10→7

k = -9→8

l = -15→15

Geometry. All esds (except the esd in the dihedral angle between two l.s. planes) are estimated using the full covariance matrix. The cell esds are taken into account individually in the estimation of esds in distances, angles and torsion angles; correlations between esds in cell parameters are only used when they are defined by crystal symmetry. An approximate

(isotropic) treatment of cell esds is used for estimating esds involving l.s. planes.

Table S4 Fractional atomic coordinates and isotropic or equivalent isotropic displacement parameters (Å^2) for compound **3**

	x	y	z	U_{iso}*/U_{eq}
O1	0.65632 (12)	0.72673 (14)	0.58568 (7)	0.0392 (3)
O2	1.00295 (11)	0.69609 (14)	0.45706 (9)	0.0429 (3)
O3	0.99434 (13)	0.54122 (19)	0.30003 (11)	0.0590 (4)
N1	0.33935 (12)	0.67988 (16)	0.33763 (9)	0.0296 (3)
N2	0.49250 (11)	0.63072 (13)	0.32195 (8)	0.0248 (2)
N3	0.50044 (13)	0.52464 (16)	0.23075 (9)	0.0357 (3)
N4	0.65883 (13)	0.50987 (16)	0.23868 (9)	0.0371 (3)
N5	0.92805 (12)	0.61435 (15)	0.36632 (10)	0.0340 (3)
N6	0.70498 (17)	0.98293 (15)	0.48681 (9)	0.0419 (3)
H6A	0.718684	1.060638	0.544251	0.050*
H6B	0.713331	1.023773	0.421711	0.050*
C1	0.74869 (14)	0.60443 (16)	0.33492 (10)	0.0277 (3)
C2	0.64521 (13)	0.68460 (15)	0.39010 (9)	0.0233 (3)
C3	0.67138 (13)	0.80234 (16)	0.49850 (9)	0.0258 (3)
H1A	0.309 (2)	0.572 (3)	0.3672 (14)	0.045 (4)*
H1B	0.271 (2)	0.704 (2)	0.2672 (16)	0.041 (4)*

Table S5 Atomic displacement parameters (Å^2) for compound **3**

	U^{11}	U^{22}	U^{33}	U^{12}	U^{13}	U^{23}
O1	0.0551 (6)	0.0394 (5)	0.0245 (4)	-0.0093 (4)	0.0145 (4)	-0.0010 (3)
O2	0.0310 (5)	0.0430 (5)	0.0464 (6)	-0.0044 (4)	0.0003 (4)	0.0013 (4)
O3	0.0358 (5)	0.0737 (8)	0.0756 (8)	0.0028 (5)	0.0291 (5)	-0.0176 (6)
N1	0.0225 (5)	0.0362 (5)	0.0304 (5)	-0.0003 (4)	0.0086 (4)	-0.0026 (4)
N2	0.0241 (5)	0.0262 (4)	0.0233 (4)	-0.0016 (3)	0.0062 (3)	-0.0040 (3)

N3	0.0318 (5)	0.0417 (6)	0.0318 (5)	-0.0010 (4)	0.0073 (4)	-0.0147 (4)
N4	0.0322 (5)	0.0435 (6)	0.0353 (6)	0.0015 (4)	0.0102 (4)	-0.0139 (4)
N5	0.0268 (5)	0.0329 (5)	0.0419 (6)	0.0012 (4)	0.0103 (4)	0.0019 (4)
N6	0.0687 (8)	0.0284 (5)	0.0279 (5)	-0.0108 (5)	0.0141 (5)	-0.0062 (4)
C1	0.0261 (5)	0.0283 (5)	0.0284 (5)	0.0003 (4)	0.0082 (4)	-0.0017 (4)
C2	0.0245 (5)	0.0224 (5)	0.0220 (5)	-0.0017 (4)	0.0059 (4)	0.0008 (4)
C3	0.0261 (5)	0.0278 (5)	0.0220 (5)	-0.0018 (4)	0.0054 (4)	-0.0020 (4)

Table S6 Geometric parameters (Å, º) for compound **3**

O1—C3	1.2198 (15)	N3—N4	1.3051 (16)
O2—N5	1.2211 (15)	N4—C1	1.3443 (15)
O3—N5	1.2209 (15)	N5—C1	1.4326 (16)
N1—N2	1.3981 (14)	N6—C3	1.3100 (17)
N1—H1A	0.905 (19)	N6—H6A	0.8600
N1—H1B	0.882 (18)	N6—H6B	0.8600
N2—C2	1.3410 (14)	C1—C2	1.3686 (15)
N2—N3	1.3469 (14)	C2—C3	1.5033 (16)
N2—N1—H1A	103.2 (11)	C3—N6—H6B	120.0
N2—N1—H1B	105.5 (11)	H6A—N6—H6B	120.0
H1A—N1—H1B	111.4 (15)	N4—C1—C2	110.70 (10)
C2—N2—N3	111.99 (9)	N4—C1—N5	120.67 (10)
C2—N2—N1	126.05 (10)	C2—C1—N5	128.62 (10)
N3—N2—N1	121.93 (9)	N2—C2—C1	102.41 (10)
N4—N3—N2	107.06 (9)	N2—C2—C3	122.63 (10)
N3—N4—C1	107.83 (10)	C1—C2—C3	134.95 (10)
O3—N5—O2	124.96 (11)	O1—C3—N6	126.44 (11)
O3—N5—C1	117.51 (11)	O1—C3—C2	119.28 (10)
O2—N5—C1	117.53 (10)	N6—C3—C2	114.25 (10)
C2—N2—N3—N4	-0.39 (14)	N3—N2—C2—C3	-179.31 (10)
N1—N2—N3—N4	177.75 (10)	N1—N2—C2—C3	2.65 (17)
N2—N3—N4—C1	0.66 (14)	N4—C1—C2—N2	0.47 (12)
N3—N4—C1—C2	-0.73 (14)	N5—C1—C2—N2	179.22 (11)

N3—N4—C1—N5	-179.59 (10)	N4—C1—C2—C3	179.58 (12)
O3—N5—C1—N4	3.06 (17)	N5—C1—C2—C3	-1.7 (2)
O2—N5—C1—N4	-177.27 (11)	N2—C2—C3—O1	74.83 (14)
O3—N5—C1—C2	-175.57 (12)	C1—C2—C3—O1	-104.14 (15)
O2—N5—C1—C2	4.09 (18)	N2—C2—C3—N6	-103.47 (13)
N3—N2—C2—C1	-0.05 (12)	C1—C2—C3—N6	77.56 (16)

Table S7 Crystal data for compounds **S8**
$C_6H_4N_{12}O_6$
M_r = 340.21
Orthorhombic, $P2_12_12_1$
a = 4.6249 (7) Å
b = 14.441 (2) Å
c = 19.195 (3) Å
V = 1282.1 (3) Å^3
Z = 4
F(000) = 688
D_x = 1.763 Mg m^{-3}
Cu $K\alpha$ radiation, λ = 1.54178 Å
Cell parameters from 2293 reflections
θ = 3.8–62.9°
μ = 1.39 mm^{-1}
T = 100 K
Needle, colourless
0.11 × 0.04 × 0.02 mm

Table S8 Refinement for compounds S8
Refinement on F^2
Least-squares matrix: full

$R[F^2 > 2\sigma(F^2)] = 0.052$
$wR(F^2) = 0.121$
$S = 1.04$
1996 reflections
217 parameters
0 restraints
Hydrogen site location: inferred from neighbouring sites
H-atom parameters constrained
$w = 1/[\sigma^2(F_o^2) + (0.0483P)^2]$ where $P = (F_o^2 + 2F_c^2)/3$
$(\Delta/\sigma)_{max} = 0.001$
$\Delta\rangle_{max} = 0.27$ e Å^{-3}
$\Delta\rangle_{min} = -0.25$ e Å^{-3}
Absolute structure: Flack x determined using 545 quotients [(I+)-(I-)]/[(I+)+(I-)] (Parsons, Flack and Wagner, Acta Cryst. B69 (2013) 249-259).
Flack parameter: -0.4 (4)

Table S9 Data collection for compounds **S8**

D8 VENTURE diffractometer
Absorption correction: multi-scan *SADABS2016*/2 (Bruker,2016/2) was used for absorption correction. wR2(int) was 0.1535 before and 0.0867 after correction. The Ratio of minimum to maximum transmission is 0.6025. The λ/2 correction factor is Not present.
$T_{min} = 0.452$, $T_{max} = 0.752$
4942 measured reflections
1996 independent reflections
1710 reflections with $I > 2\sigma(I)$
$R_{int} = 0.080$

θ_{max} = 63.7°, θ_{min} = 3.8°
h = -4→5
k = -16→16
l = -22→17

Geometry. All esds (except the esd in the dihedral angle between two l.s. planes) are estimated using the full covariance matrix. The cell esds are taken into account individually in the estimation of esds in distances, angles and torsion angles; correlations between esds in cell parameters are only used when they are defined by crystal symmetry. An approximate (isotropic) treatment of cell esds is used for estimating esds involving l.s. planes.

Table S10 Fractional atomic coordinates and isotropic or equivalent isotropic displacement parameters (Å²) for compound **S8**

	x	*y*	*z*	U_{iso}*/U_{eq}
O4	1.2943 (9)	1.5499 (2)	1.56007 (18)	0.0304 (9)
O6	1.6996 (10)	1.7411 (2)	1.3714 (2)	0.0385 (11)
O3	1.2717 (9)	1.3346 (3)	1.68987 (17)	0.0329 (9)
O2	1.5528 (10)	1.1441 (2)	1.74888 (18)	0.0355 (10)
O1	1.9019 (10)	1.0528 (2)	1.71651 (18)	0.0359 (10)
O5	1.4362 (10)	1.7215 (2)	1.46443 (19)	0.0342 (10)
N2	1.9970 (10)	1.1617 (3)	1.60170 (19)	0.0248 (10)
N10	1.6208 (11)	1.6969 (3)	1.4217 (2)	0.0269 (11)
N7	1.8806 (10)	1.4778 (2)	1.47201 (19)	0.0210 (10)
N5	1.7928 (10)	1.3845 (3)	1.55659 (19)	0.0211 (10)
N8	2.0043 (11)	1.4880 (2)	1.40853 (19)	0.0242 (11)
N12	1.6970 (11)	1.3912 (3)	1.7311 (2)	0.0270 (10)
H12A	1.613884	1.431031	1.759441	0.032*
H12B	1.886736	1.387846	1.728984	0.032*
N4	1.8400 (10)	1.2989 (3)	1.5859 (2)	0.0215 (10)
N1	1.7552 (11)	1.1226 (3)	1.7103 (2)	0.0262 (11)
N11	1.7161 (11)	1.5900 (3)	1.6120 (2)	0.0251 (10)

H11A	1.636325	1.599187	1.653074	0.030*
H11B	1.903162	1.598384	1.606475	0.030*
N6	1.9404 (10)	1.3934 (3)	1.50269 (19)	0.0217 (9)
N9	1.9231 (11)	1.5681 (3)	1.3850 (2)	0.0262 (10)
N3	2.0123 (10)	1.2306 (3)	1.5586 (2)	0.0232 (10)
C1	1.8157 (12)	1.1852 (3)	1.6547 (2)	0.0203 (12)
C4	1.7183 (12)	1.5520 (3)	1.4910 (2)	0.0227 (11)
C5	1.5559 (13)	1.5639 (3)	1.5590 (2)	0.0234 (11)
C2	1.7134 (12)	1.2730 (3)	1.6467 (2)	0.0201 (11)
C6	1.7501 (12)	1.6070 (3)	1.4341 (2)	0.0230 (12)
C3	1.5375 (13)	1.3368 (3)	1.6918 (2)	0.0224 (11)

Table S11 Atomic displacement parameters (Å^2) for **S8**.

	U^{11}	U^{22}	U^{33}	U^{12}	U^{13}	U^{23}
O4	0.018 (2)	0.0354 (19)	0.038 (2)	-0.0015 (18)	-0.0022 (19)	-0.0030 (16)
O6	0.046 (3)	0.0301 (19)	0.039 (2)	0.0077 (19)	0.006 (2)	0.0097 (17)
O3	0.016 (3)	0.042 (2)	0.041 (2)	-0.0003 (19)	0.0001 (19)	-0.0091 (17)
O2	0.038 (3)	0.0374 (19)	0.0311 (18)	-0.0013 (19)	0.0107 (19)	0.0035 (16)
O1	0.035 (3)	0.0314 (18)	0.041 (2)	0.002 (2)	-0.0034 (18)	0.0080 (17)
O5	0.031 (3)	0.0313 (19)	0.040 (2)	0.0094 (19)	0.002 (2)	0.0015 (16)
N2	0.024 (3)	0.0254 (19)	0.025 (2)	-0.003 (2)	-0.0024 (19)	0.0004 (17)
N10	0.023 (3)	0.028 (2)	0.029 (2)	0.005 (2)	-0.002 (2)	0.0003 (18)
N7	0.020 (3)	0.0205 (19)	0.0225 (19)	0.0001 (18)	0.0015 (19)	0.0002 (15)
N5	0.020 (3)	0.0208 (19)	0.023 (2)	-0.0004 (18)	-0.003 (2)	-0.0012 (16)
N8	0.025 (3)	0.0238 (19)	0.024 (2)	0.000 (2)	0.001 (2)	0.0016 (16)
N12	0.016 (3)	0.032 (2)	0.034 (2)	0.000 (2)	0.001 (2)	-0.0125 (18)
N4	0.019 (3)	0.0206 (19)	0.025 (2)	0.0019 (17)	-0.0011 (18)	-0.0007 (16)
N1	0.027 (3)	0.026 (2)	0.026 (2)	-0.002 (2)	-0.004 (2)	0.0001 (17)
N11	0.020 (3)	0.030 (2)	0.026 (2)	-0.0027 (19)	0.003 (2)	-0.0035 (16)
N6	0.017 (2)	0.026 (2)	0.021 (2)	-0.001 (2)	-0.001 (2)	-0.0001 (16)
N9	0.027 (3)	0.025 (2)	0.026 (2)	0.003 (2)	0.001 (2)	-0.0003 (17)

N3	0.021 (3)	0.0226 (19)	0.026 (2)	0.0016 (18)	0.0024 (19)	-0.0027 (16)
C1	0.017 (3)	0.025 (2)	0.018 (2)	-0.002 (2)	-0.002 (2)	-0.0014 (18)
C4	0.015 (3)	0.027 (2)	0.026 (2)	-0.002 (2)	-0.004 (2)	-0.005 (2)
C5	0.023 (3)	0.021 (2)	0.026 (3)	0.001 (2)	-0.002 (3)	-0.001 (2)
C2	0.012 (3)	0.028 (2)	0.021 (2)	-0.004 (2)	-0.002 (2)	-0.0031 (19)
C6	0.018 (3)	0.024 (2)	0.027 (2)	0.002 (2)	-0.003 (2)	-0.002 (2)
C3	0.018 (3)	0.023 (2)	0.027 (2)	0.001 (2)	0.000 (2)	0.001 (2)

Table S12 Geometric parameters (Å, º) for **S8**.

O4—C5	1.227 (7)	N12—C3	1.315 (7)
O6—N10	1.215 (6)	N12—H12A	0.8800
O3—C3	1.230 (7)	N12—H12B	0.8800
O2—N1	1.233 (6)	N4—C2	1.358 (6)
O1—N1	1.221 (6)	N4—N3	1.372 (6)
O5—N10	1.236 (6)	N1—C1	1.427 (6)
N2—N3	1.297 (5)	N11—C5	1.314 (7)
N2—C1	1.361 (7)	N11—H11A	0.8800
N10—C6	1.448 (6)	N11—H11B	0.8800
N7—N8	1.354 (5)	N9—C6	1.358 (6)
N7—C4	1.358 (6)	C1—C2	1.361 (7)
N7—N6	1.382 (5)	C4—C6	1.359 (6)
N5—N6	1.246 (6)	C4—C5	1.517 (7)
N5—N4	1.375 (5)	C2—C3	1.504 (7)
N8—N9	1.298 (5)		
N3—N2—C1	108.5 (4)	N5—N6—N7	109.6 (4)
O6—N10—O5	125.7 (4)	N8—N9—C6	107.3 (4)
O6—N10—C6	118.5 (4)	N2—N3—N4	106.1 (4)
O5—N10—C6	115.8 (4)	C2—C1—N2	111.3 (4)
N8—N7—C4	112.9 (4)	C2—C1—N1	127.3 (5)
N8—N7—N6	113.3 (4)	N2—C1—N1	121.4 (4)
C4—N7—N6	133.8 (4)	N7—C4—C6	100.7 (4)

N6—N5—N4	110.2 (4)	N7—C4—C5	126.4 (4)
N9—N8—N7	106.8 (4)	C6—C4—C5	132.8 (5)
C3—N12—H12A	120.0	O4—C5—N11	126.2 (5)
C3—N12—H12B	120.0	O4—C5—C4	118.9 (5)
H12A—N12—H12B	120.0	N11—C5—C4	114.8 (5)
C2—N4—N3	112.4 (4)	N4—C2—C1	101.8 (4)
C2—N4—N5	122.1 (4)	N4—C2—C3	124.0 (4)
N3—N4—N5	125.6 (4)	C1—C2—C3	133.9 (4)
O1—N1—O2	124.8 (4)	N9—C6—C4	112.3 (4)
O1—N1—C1	119.2 (5)	N9—C6—N10	120.0 (4)
O2—N1—C1	116.0 (4)	C4—C6—N10	127.7 (5)
C5—N11—H11A	120.0	O3—C3—N12	126.4 (5)
C5—N11—H11B	120.0	O3—C3—C2	120.5 (5)
H11A—N11—H11B	120.0	N12—C3—C2	113.2 (5)
C4—N7—N8—N9	-0.5 (6)	C6—C4—C5—N11	-99.3 (7)
N6—N7—N8—N9	177.9 (4)	N3—N4—C2—C1	-0.4 (6)
N6—N5—N4—C2	177.4 (4)	N5—N4—C2—C1	179.1 (4)
N6—N5—N4—N3	-3.1 (7)	N3—N4—C2—C3	173.6 (5)
N4—N5—N6—N7	177.2 (4)	N5—N4—C2—C3	-6.8 (8)
N8—N7—N6—N5	-172.1 (4)	N2—C1—C2—N4	1.0 (6)
C4—N7—N6—N5	5.9 (7)	N1—C1—C2—N4	179.9 (5)
N7—N8—N9—C6	0.1 (6)	N2—C1—C2—C3	-172.2 (6)
C1—N2—N3—N4	0.8 (6)	N1—C1—C2—C3	6.7 (10)
C2—N4—N3—N2	-0.3 (6)	N8—N9—C6—C4	0.4 (6)
N5—N4—N3—N2	-179.7 (4)	N8—N9—C6—N10	-178.8 (5)
N3—N2—C1—C2	-1.2 (6)	N7—C4—C6—N9	-0.7 (6)
N3—N2—C1—N1	179.8 (4)	C5—C4—C6—N9	178.8 (5)
O1—N1—C1—C2	-167.2 (5)	N7—C4—C6—N10	178.4 (5)
O2—N1—C1—C2	13.1 (8)	C5—C4—C6—N10	-2.2 (10)
O1—N1—C1—N2	11.7 (7)	O6—N10—C6—N9	-9.4 (7)
O2—N1—C1—N2	-168.1 (5)	O5—N10—C6—N9	170.5 (5)
N8—N7—C4—C6	0.7 (6)	O6—N10—C6—C4	171.6 (5)
N6—N7—C4—C6	-177.3 (5)	O5—N10—C6—C4	-8.5 (8)

N8—N7—C4—C5	-178.7 (5)	N4—C2—C3—O3	100.3 (6)
N6—N7—C4—C5	3.2 (9)	C1—C2—C3—O3	-87.8 (8)
N7—C4—C5—O4	-100.9 (6)	N4—C2—C3—N12	-79.8 (6)
C6—C4—C5—O4	79.8 (8)	C1—C2—C3—N12	92.1 (7)
N7—C4—C5—N11	80.0 (6)		

3. ^{1}H and ^{13}C NMR spectra for all new compounds

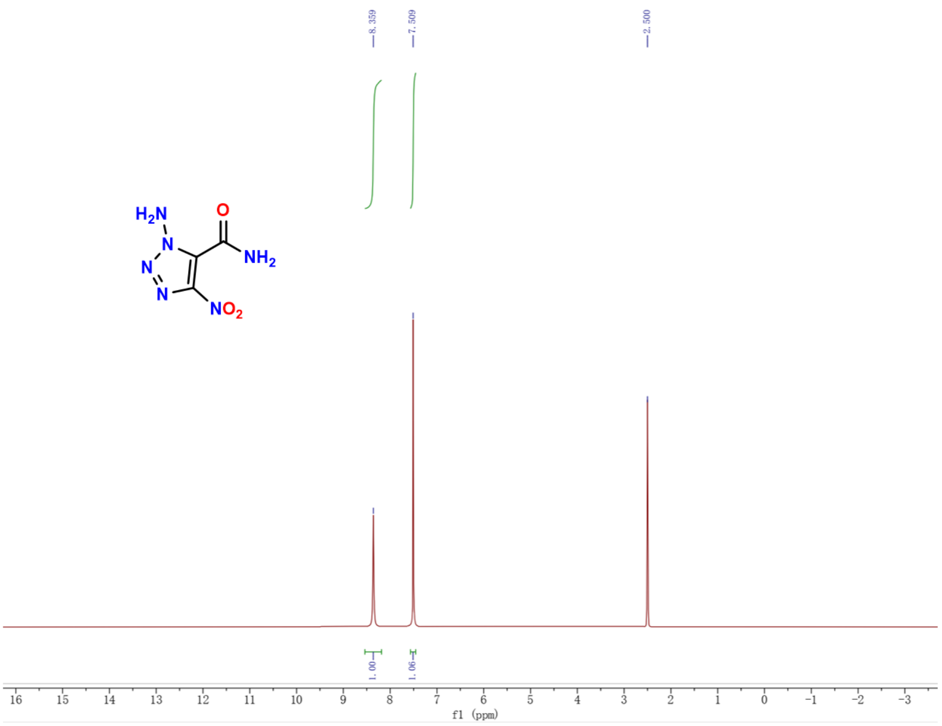

Figure S2. ^{1}H NMR spectrum of compound **3** in DMSO-d_6.

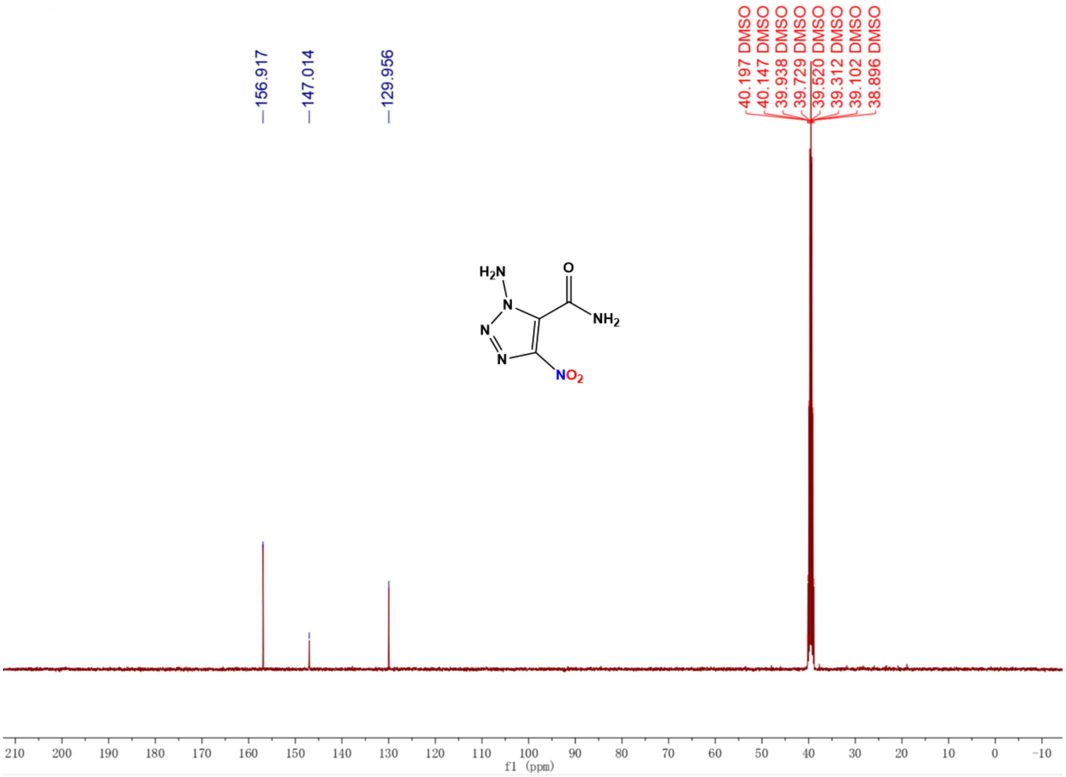

Figure S3. ^{13}C NMR spectrum of compound **3** in DMSO-d_6.

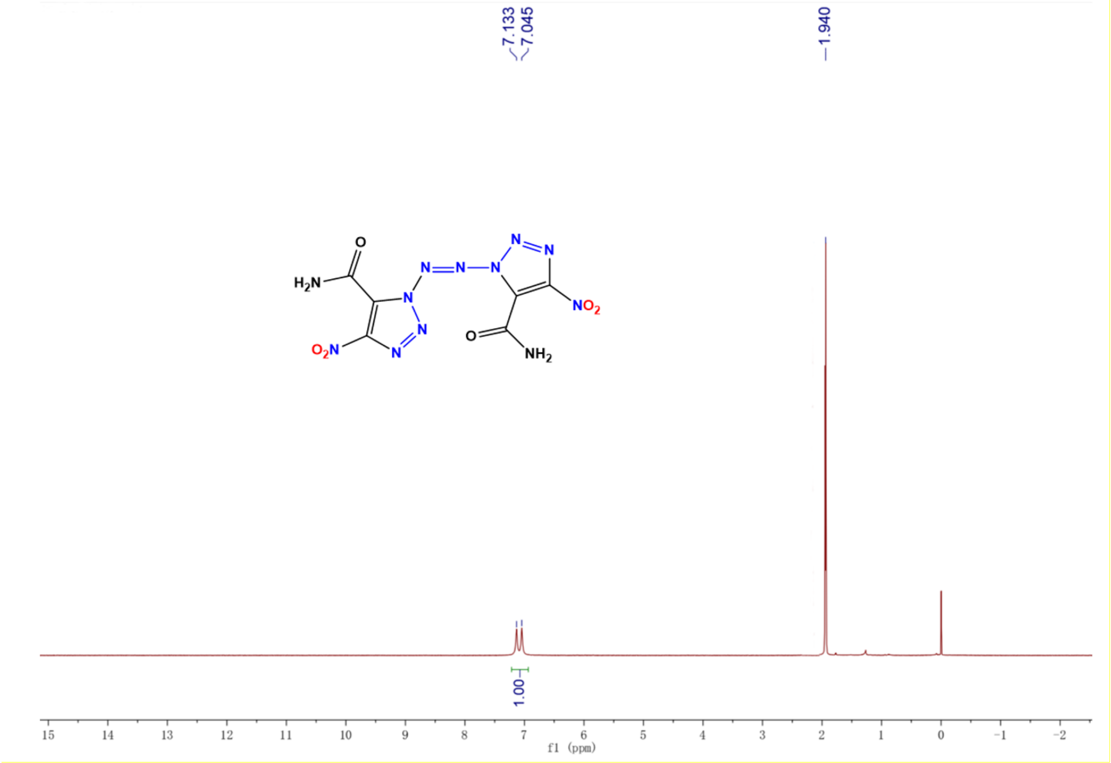

Figure S4. ^{1}H NMR spectrum of **S8** in d_3-CD_3CN.

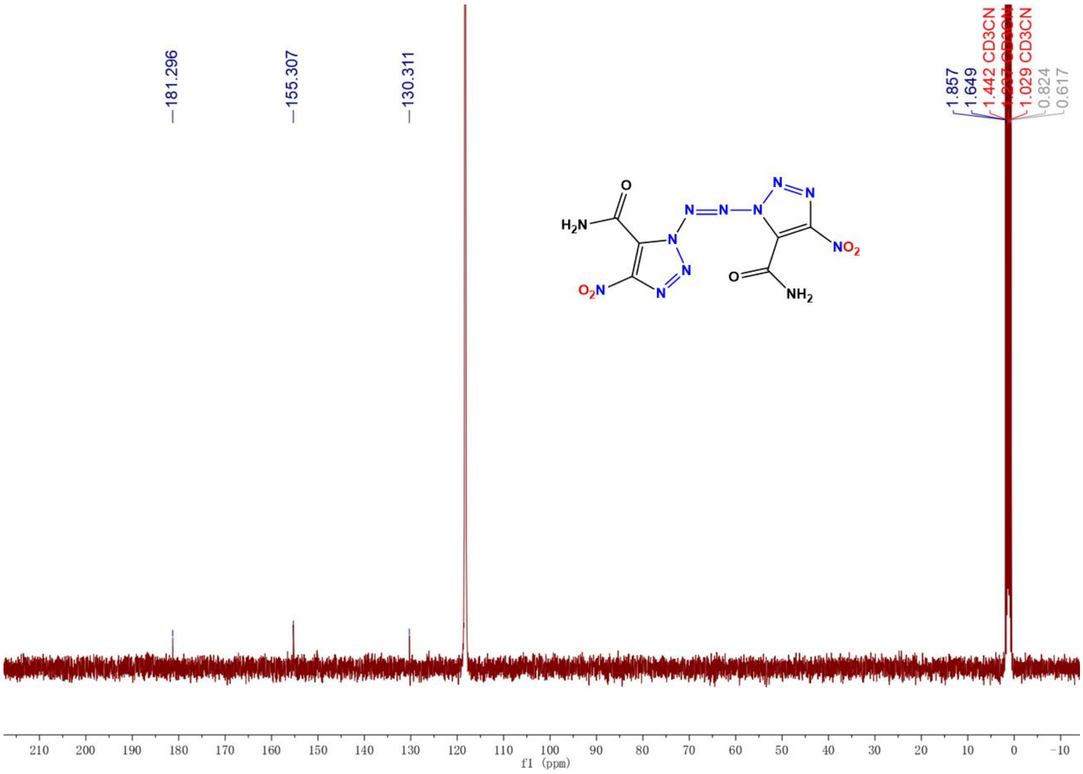

Figure S5. ^{13}C NMR spectrum of **S8** in d_3-CD_3CN.

4. IR spectra of all new compounds

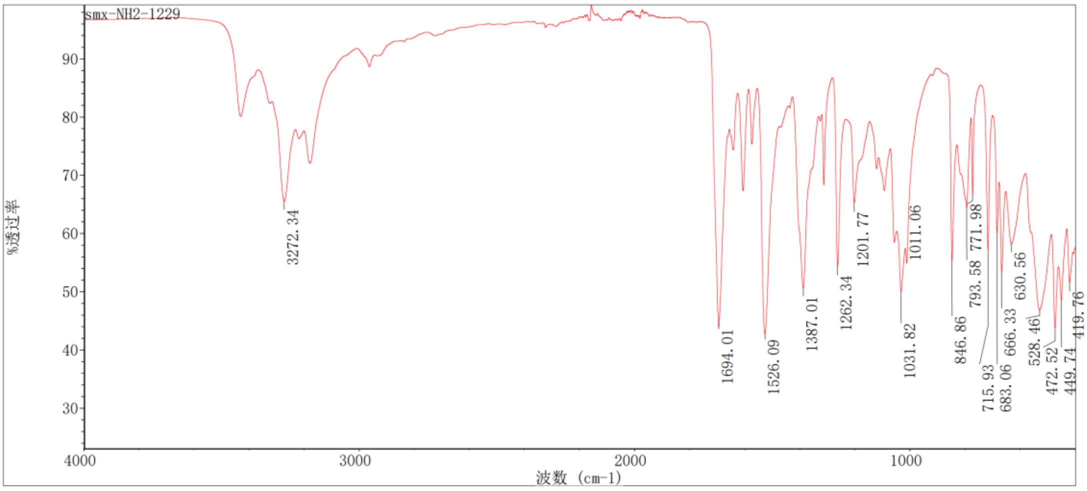

Figure S6. IR spectrum of compound 3.

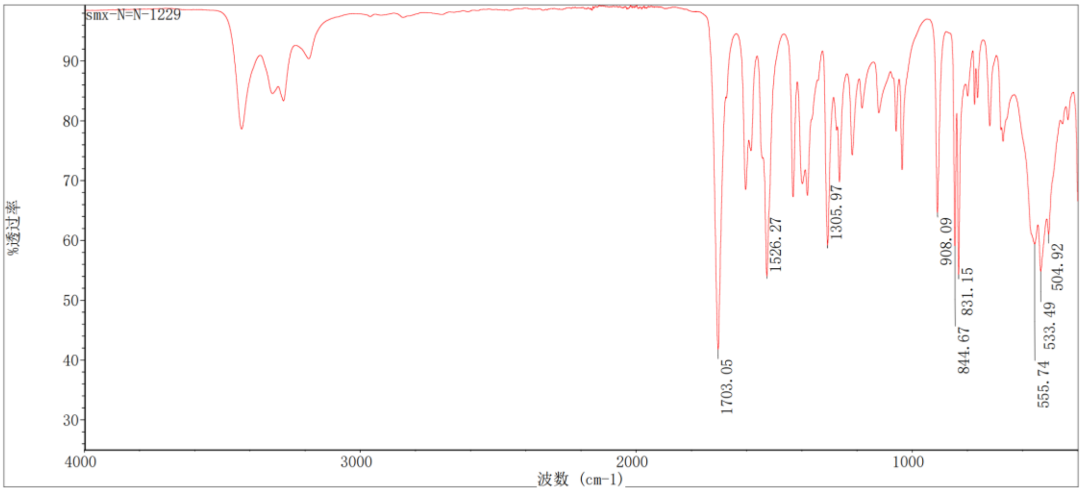

Figure S7. IR spectrum of compound **S8**

4. DSC curves of the title compounds

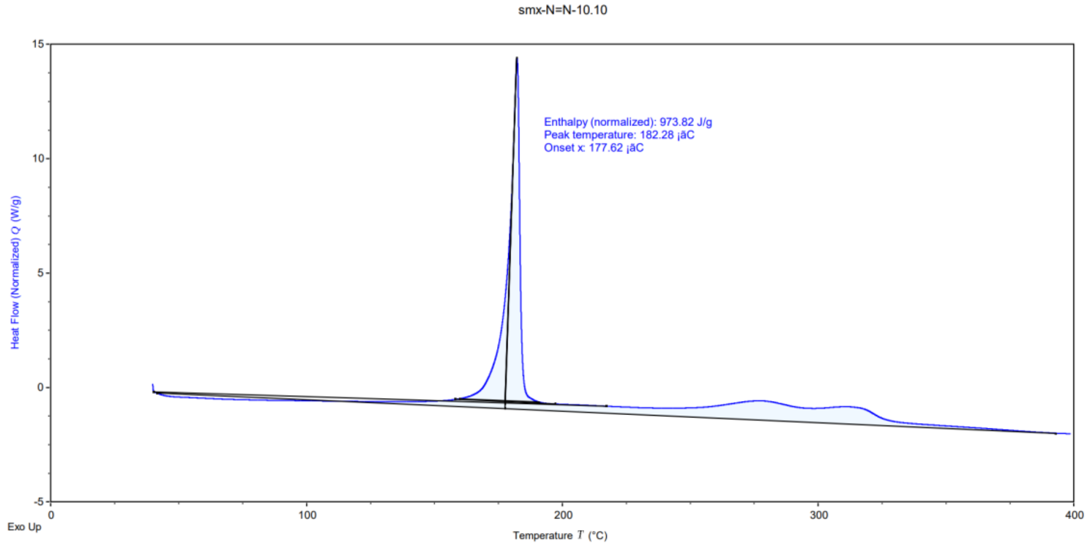

Figure S8. DSC curve of compound **S8** at 10 °C min^{-1}.

References

(1) Schawartz, J., Hornyák, M. and Süts, T., Crystal structure of 5-amino-1H-1,2,3-triazole-4-carboxamide, *J. Chem. Soc., Perkin Trans. 2*, **1974**, *1849*.
(2) Becke, A. D., Density-functional thermochemistry. III. The role of exact exchange, *J. Phys. Chem.*, **1993**, *98*, 5648;
(3) Stephens, P. J., Devlin, F. J., Chabalowski, C. F. and Frisch, M. J., Ab Initio Calculation of Vibrational Absorption and Circular Dichroism Spectra Using Density Functional Force Fields, *J. Phys. Chem.*, **1994**, *98*, 11623.
(4) Westwell, M. S., Searle, M. S. and Williams, D. H., Empirical Correlations between Thermodynamic Properties and Intermolecular Forces, *J. Am. Chem. Soc.*, **1995**, *117*, 5013.
